# Deriving breeding goals and expected selection responses to reduce environmental impacts in rainbow trout farming

**DOI:** 10.1186/s12711-025-01018-5

**Published:** 2025-12-13

**Authors:** Simon Pouil, Joël Aubin, Florence Phocas

**Affiliations:** 1 Université Paris-Saclay, INRAE, AgroParisTech, GABI, Jouy-en-Josas, France; 2 INRAE, Institut Agro, SAS, Rennes, France

## Abstract

**Background:**

With growing societal concerns about the sustainability of food production systems, there is increasing interest in considering not only economic gains but also environmental issues in breeding programs of farmed species. In this study, we compared expected selection responses for breeding programs aiming to minimize environmental impacts of the production of rainbow trout in France, one of the most important fish species in salmonid aquaculture. The consequences of genetic improvement based on environmental merit indices were investigated in a hypothetical rainbow trout production farm with a constant annual production of 300 tonnes of fish. The merit indices included three different traits: thermal growth coefficient (TGC), daily feed intake (DFI), and survival (SR). A cradle-to-farm-gate life cycle assessment was conducted to evaluate the environmental values of each trait, which served as weightings in breeding goals aiming at minimizing expected environmental impacts by genetic selection. We explored nine different environmental impact categories: climate change, terrestrial acidification, freshwater eutrophication, marine eutrophication, terrestrial ecotoxicology, freshwater ecotoxicology, land use, water dependence, and cumulative energy demand.

**Results:**

Selection accuracy ranged from 0.34 to 0.43, with the lowest accuracy observed for the breeding goal targeting reduced water dependence, and the highest for those targeting reductions in eutrophication and terrestrial ecotoxicity. Annual genetic gains in reductions of environmental impacts, expressed per tonne of trout, were high for reducing eutrophication potential (− 6.80 to − 2.61%) and terrestrial ecotoxicity (− 4.14 to − 1.59%), but negligible for water use reduction (− 0.04 to − 0.01%). Genetic changes in DFI and TGC led to substantial annual gains in feed conversion ratio, from 1.7 to 4.8%. However, SR showed no improvement and often declined, highlighting the difficulty of balancing genetic gains across traits.

**Conclusions:**

We demonstrated the benefits of using environmental values in breeding goals to minimize environmental impacts at the farm level, while maintaining high genetic gains in feed efficiency traits. Nevertheless, we also showed that selection efficiency was highly dependent of the impact category. Our results suggested that another selection strategy should be considered to avoid unfavourable consequences on SR.

**Supplementary Information:**

The online version contains supplementary material available at 10.1186/s12711-025-01018-5.

## Background

In animal breeding, selection typically targets multiple traits, even when the primary objective focuses on a dominant production trait. Since Hazel [1] introduced the total merit index, derived to maximize selection response on a linear multi-trait target function named ‘aggregate genotype’, the methodology for defining breeding goals has been debated [2–5]. Breeding goal traits are often weighted based on desired gains in breeding programs for fish [6, 7] and poultry [8], while nowadays, weights are more frequently based on economic values (EV) in breeding goals for pigs [9, 10] and ruminants [11–13]. These EV are commonly estimated as the change in farm profit (derived from a bio-economic model of a hypothetical farm) due to a one physical unit change in the average value of a trait keeping all other traits constant [2]. In other words, the EV of each trait reflects its marginal profit contribution per unit of trait change [14]. In selection index theory, the optimal weights on the phenotype or estimated breeding value for each trait in a multi-trait linear merit index are derived to maximize the expected selection response in the breeding goal [1, 14]. Using this optimal selection index directs the selection response in each trait to maximize the total economic returns from genetic improvement. However, EV may not be the most suitable weights for enhancing the environmental and social sustainability of animal breeding programs [15].

Beyond economic values, several methodological approaches have been developed to integrate non-market or social priorities into breeding goals. One option is the desired gains approach, where breeders define acceptable trade-offs between production and environmental traits [9, 16, 17]. Another strategy is to rely on contingent valuation and choice experiments, which estimate consumers’ willingness-to-pay for improvements in sustainability or welfare traits [15, 18]. More recently, participatory and multi-criteria decision-making methods [19], such as analytic hierarchy processes, have been applied to aquaculture, including rainbow trout, to derive consensus on trait priorities among farmers with diverse objectives [6]. Comprehensive reviews also emphasize the importance of incorporating such non-market values into breeding objectives [20, 21]. These approaches illustrate how commercial breeding programs can go beyond strict profitability and explicitly account for environmental and social values, thereby aligning with broader sustainability goals.

With rising societal concern regarding the environmental sustainability of animal production, there is increasing interest in balancing economic gains with environmental impacts when defining breeding goals and selection programs. Although research on sustainable breeding goals is still limited, recent studies in cattle [22, 23], pigs [24], and fish [25, 26] have started addressing these concerns, particularly by estimating environmental values (ENV) of traits of interest. Wall et al. [27] suggested to calculate ENV based on the principle of EV from Hazel [1] asthe difference in environmental impacts between a base situation and a situation with genetic improvement in one trait, while keeping the other traits constant. Thus, to effectively integrate ENV into breeding goals, it is essential to first quantify the environmental impacts of specific traits at the farm level. Life-cycle assessment (LCA) is an internationally standardized, comprehensive method for evaluating the environmental impact of a product or a service [28] and provides a robust framework to derive ENV. The LCA method encompasses every stage of a product’s life cycle, from raw material acquisition and energy consumption to manufacturing, transportation, emissions, and disposal. By applying LCA, ENV can be derived for specific traits, which can then be integrated into an environmental merit index that minimizes the expected environmental impacts of genetic selection (i.e. maximize the reduction of the environmental impacts associated with genetic improvement of an animal population).

To illustrate the application of ENV in defining sustainable breeding goals, Besson et al. [25] conducted a study on European seabass (*Dicentrarchus labrax*). They compared the use of EV, calculated as monetary gain per one unit of trait change, versus the use of ENV, calculated as the changes in eutrophication potential per one unit of trait change, to define a breeding goal consisting of two important production traits, the thermal growth coefficient (TGC) and the feed conversion ratio (FCR). Under weak genetic correlations between the two traits (from − 0.4 to 0), the authors found that using ENV led to annual reductions in eutrophication ranging from 0.9% to 2.7% per ton of fish produced, whereas using EV resulted in smaller reductions, ranging from 0.6% to 1.5%. These findings underscore the potential of ENV to drive meaningful environmental improvements in fish production systems.

Rainbow trout (*Oncorhynchus mykiss*) is a worldwide farmed salmonid species in fresh and cold waters (1,004,000 tons in 2022 [29]). In France, rainbow trout is mainly produced in flow-through systems that divert river water through raceway tanks and release it to the river. Rainbow trout farming depends on formulated feed that contains fish meal, fish oil, and plant-based ingredients. Production targets vary, ranging from fry to large trout for different markets (e.g., pan-sized trout, fillets, or fish for stocking [30]). This diversity in production practices and objectives drives breeding programs, which have traditionally focused on productivity (growth, processing yield, disease resistance) and consumer preferences (morphology, product quality [31]). Over the past two decades, LCA has been widely applied to assess the environmental impacts of salmonid production, with numerous studies specifically examining the optimization of farming practices, feed formulation, and resource management in rainbow trout production [30, 32–37].

In this context, our study aimed to (i) define various breeding goals based on ENV derived from different LCA impact categories and (ii) predict the expected selection responses for these breeding goals and for the three key traits of interest (growth rate, feed intake, and survival) in a fish farm producing large rainbow trout.

## Methods

### Farm design and operations

The farm model, developed using the R freeware [38], was based on the model described in Pouil et al. [36]. In the present study, we simulated the conventional production of large rainbow trout in a hypothetical farm, using actual farm data obtained from surveys conducted in February 2022 in Brittany, one of the leading regions for trout production in France [39]. The flow-through production system consisted of 12 concrete raceways of 100 m^3^ for the pre-growing phase and 24 concrete raceways of 250 m^3^ for grow-out. Among the 250 m^3^ raceways, 50% received first water, meaning that the water entered the tanks directly from the river by gravity, while the remaining 50% received second water, i.e. the outlet water from the upstream raceways. The average monthly temperature varied from 5 to 21 °C throughout the year, while the water flow ranged from 0.33 to 1.5 m^3^ s^− 1^. During low water flow periods, from May to September, a 1-kWh drum filter and a 20-kWh pump operated to ensure effective water recirculation. Liquid oxygen was used to maintain an O_2_ saturation level of 80% at the outlet of the raceways.

Fish were initially stocked at 10 g and harvested at a fixed weight of 3000 g. The maximal annual production was fixed at 300 tonnes. Three batches of fry were stocked in January, May, and August to stagger the sales period. As the fish grew, they were periodically redistributed into 2 and then 4 raceways of 100 m^3^ before ultimately occupying 4, and then 8 raceways of 250 m^3^. The maximum density constraints varied depending on the production scenario and were 50 kg m^− 3^ when weight was less than 50 g, 70 kg m^− 3^ when weight ranged from 50 to 1000 g, and 90 kg m^− 3^ when weight was greater than 1000 g. Over the production cycle (i.e. 24 months), fish were fed using four different diets and their rationing was adjusted according to fish size and water temperature.

## Life cycle assessment

### Goal and scope

**Fig. 1:**
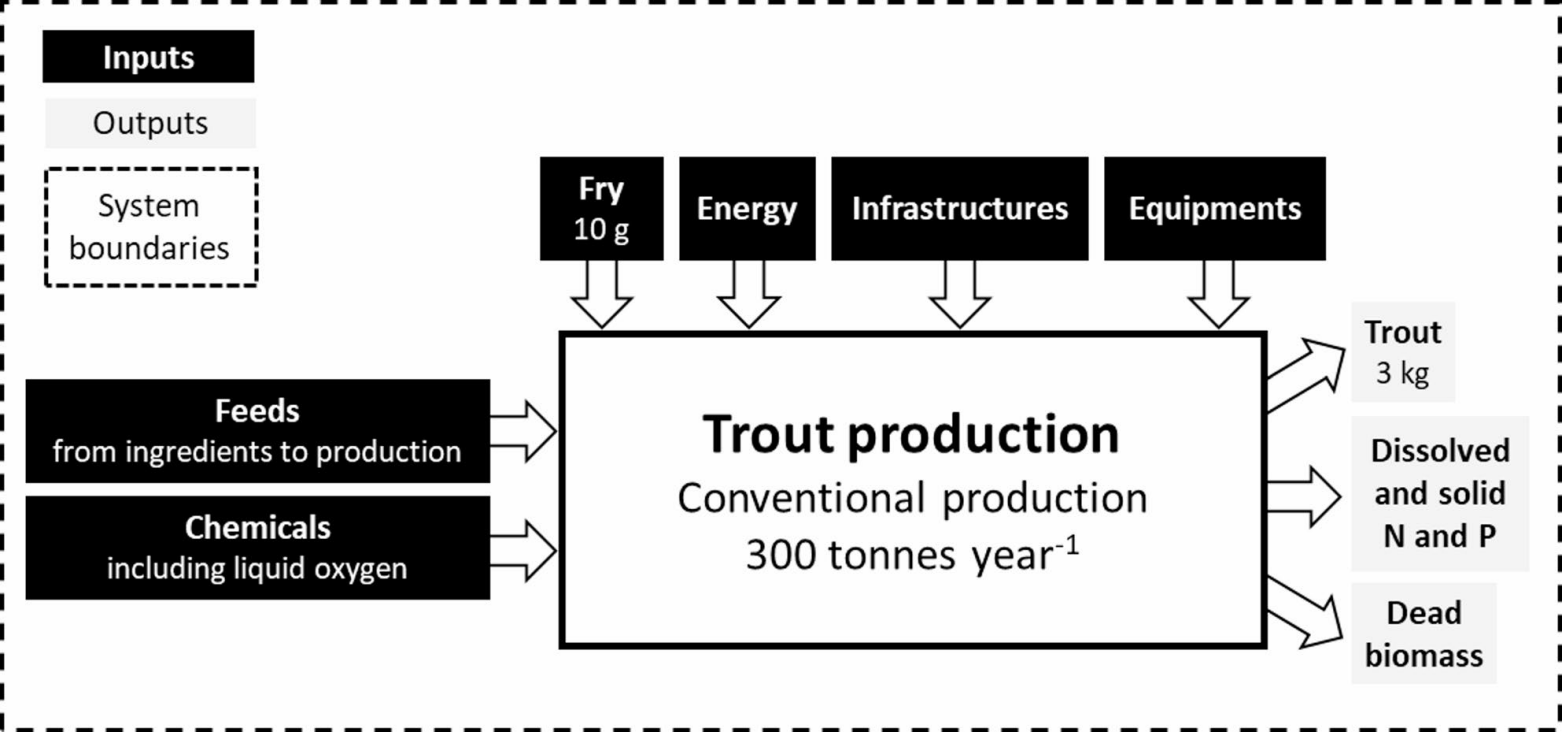
System boundaries and flows in conventional production of rainbow trout *Oncorhynchus mykiss*. The system boundaries are represented by dashed lines, while the inputs and outputs of the production system are highlighted in black and grey, respectively

A LCA was conducted according to the general requirements of the methodology proposed by ILCD standards [40]. The goal and scope of this study was the environmental assessment of trout farming in a hypothetical farm producing only large rainbow trout. The environmental impacts of changes in the performances of several fish traits (see Section Traits for details) were investigated. The system was defined from cradle-to-farm-gate and included distinct sub-systems (Fig. 1): production of 10 g fry; production of purchased feed, including cultivation of ingredients, processing, and transportation; production of energy expended at farm level (electricity); production of farming facilities and equipment used; chemicals, including liquid oxygen, medicines, and disinfectants, and their transportation; fish farming, including nutrient emissions from the biological transformation of feed after onsite treatment of wastewater and dead biomass. The functional unit on which environmental impacts were calculated was the production of one tonne of trout of market size (3000 g) at farm level.

## Life cycle inventory

The life cycle inventory was performed as described by Pouil et al. [36], using the farm model specifications for conventional production. Inputs and outputs were calculated based on annual data from routine production. The Agribalyse version 3.0 [41] and Ecoinvent version 3.8 [42] databases provided necessary data, following international standards [43].

Feed production - Crop-derived ingredients mainly came from Brazil (e.g., soybean meal) and France (e.g., wheat bran), while fish-derived ingredients originated from Peruvian and Norwegian fish milling industries. Feed composition and nutritional values were provided by manufacturers. Transport involved trans-oceanic shipping and lorry (> 32 t), with distances estimated via Google Maps and shiptraffic.net.

Energy use - Electricity was sourced from the French energy mix (Ecoinvent database), while diesel and gas consumption were negligible.

Farming facilities & equipment - The model included two buildings (30-year lifespan) and equipment (pumps, tanks), using data from INRAE. Usage was adjusted for seasonal and fish size variations.

Chemicals - Chemicals included disease treatments (antibiotics, veterinary, disinfection products) and liquid oxygen production from cryogenic air separation.

Farm operations - This encompassed energy use, facility and equipment operation, and pollutant emissions from feed metabolism. Emissions (N, P, COD) in effluent water were calculated using a mass balance approach, following Pouil et al. [36], considering sludge pond treatment capacity. Farm sludge was repurposed for agriculture and was, therefore, excluded from the analysis.

Dead biomass - This represented all dead biomass accumulated during production, taking its incineration into account.

## Life cycle impact assessment

The assessment of the environmental impacts was carried out using the ReCiPe 2016 Midpoint (H) version 1.07 software [44], which, represents the most up-to-date and standardized indicator approach available for LCA [40]. We selected the nine impact categories from ReCiPe Midpoint that have been identified among the most suitable indicators of aquaculture impacts [45], namely climate change (kg CO_2_ eq), terrestrial acidification potential (kg SO_2_ eq), freshwater eutrophication (kg P eq), marine eutrophication (kg N eq), terrestrial ecotoxicology (kg 1,4-DCB), freshwater ecotoxicology (kg 1,4-DCB), land use (m^2^ yr^− 1^ crop eq), completed by water dependence (m^3^) [26], and Cumulative Energy Demand (CED in GJ [46]). The environmental impacts were calculated using the Simapro version 8.0 software.

### Breeding scheme design

We modelled a straightforward breeding program using R freeware version 4.2.2, inspired by current breeding schemes applied for rainbow trout selection in France. The deterministic model used to predict selection responses assumed an equal number of male and female breeders (*n* = 100 for each sex), discrete generations, and a reproductive age for both sexes of 24 months. The generation interval was therefore 24 months for both sexes. Candidate selection (*n* = 1000 for each sex) was based on own performance for growth and feed intake, while survival was assessed through a collateral test conducted on half-sibs in a controlled testing station with a capacity of 1200 individuals. We assumed that all selection candidates had 23 half-sibs tested. Each generation, 10% of the candidates (*n* = 100 for each sex) were selected of brooders for the next generation.

### Traits

We considered three traits to be under selection, covering key aspects of trout production: growth rate, feed intake, and survival rate. These traits were identified as priorities by farmers in previous surveys and studies on breeding preferences in rainbow trout (e.g., Sae-Lim et al. [6]). For each LCA environmental impact category (X), we defined a specific breeding goal (H_X_), expressed as a weighted combination of the breeding values of these three traits based on their environmental values (ENV), as described in the “Breeding goals” section.

In trout farming, the market is segmented, with products primarily sold at specific weights, while regulatory constraints limit farm production capacity. As a result, growth rate is a critical factor, as faster growth can shorten production cycles. To address this, the thermal growth coefficient (TGC) was selected as the first trait in the breeding goal. Given the non-linear relationship between growth rate and water temperature, as proposed by Besson et al. [47] and used by Pouil et al. [36], the TGC formula was adjusted to account for the concave growth-temperature relationship by using a corrected temperature factor K, calculated at a given day (n) as:

1 $$\:{\text{K}}_{\text{n}}=\:\frac{{\text{T}}_{\text{o}\text{p}\text{t}}\left({\text{T}}_{\text{n}}-{\text{T}}_{\text{m}\text{i}\text{n}}\right)\left({\text{T}}_{\text{n}}-{\text{T}}_{\text{m}\text{a}\text{x}}\right)}{\left({\text{T}}_{\text{n}}-{\text{T}}_{\text{m}\text{i}\text{n}}\right)\left({\text{T}}_{\text{n}}-{\text{T}}_{\text{m}\text{a}\text{x}}\right){\:-\:\left({\text{T}}_{\text{n}}-{\text{T}}_{\text{o}\text{p}\text{t}}\right)}^{2}},$$

for T_min_ ≤ K_n_ ≤ T_max_ and K = 0 for other values. Here, T_min_ and T_max_ represent the minimum and maximum temperatures, respectively, below and above which growth does not occur. T_opt_ refers to the optimal temperature for growth. Based on extrapolations from Bear et al. [48], the values for rainbow trout were set at 3 °C for T_min_ (K_n_ = 0), 13 °C for T_opt_ (K_n_ = 13), and 24 °C for T_max_ (K_n_ = 0). Consequently, T_n_ must fall between 3 °C and 24 °C for a positive growth rate. TGC on day n was then calculated following:

2 $$\:{\text{T}\text{G}\text{C}}_{n}=\:\frac{{\text{W}}_{\text{H}}^{\text{b}}-{\text{W}}_{\text{I}}^{\text{b}}}{{\sum\:}_{\text{i}=1}^{\text{n}}{\text{K}}_{\text{i}}}\times1000,$$

where W_H_ represents the final weight at harvest (3000 g), W_I_ denotes the initial weight at stocking (10 g), n is the rearing time in days, and b is a weight coefficient, which was set at 1/3 for the overall growing period [49]. In the reference scenario [36], the mean TGC was equal to 1.8 g^1/3^ d^− 1^ °C^− 1^, such that harvest weight was achieved in 737 days on average.

Feed efficiency (FE) is a key determinant of both economic performance and environmental impacts in aquaculture. FE is often measured through ratio traits such as feed conversion ratio (FCR), but direct selection on ratio traits is problematic because it does not allow control over whether genetic gain comes from the numerator (growth) or the denominator (feed intake), and can lead to undesirable correlated responses, as highlighted in classical selection index theory [1, 50]. A more robust strategy is to decompose FE into its component traits and include them in a linear selection index. Lin [51], in a study on mice, showed that an index combining body weight gain and feed intake provided greater and more predictable improvement in FE than direct selection on the ratio. Following this rationale, daily feed intake (DFI) was the second trait included in the breeding goal. This modelling approach allows both direct and correlated responses in FCR to be predicted, while avoiding the pitfalls of ratio-based selection.

DFI was calculated following a bioenergetic approach [52] as:

3 $$\:\text{D}\text{F}\text{I}=\:\frac{\text{D}\text{E}}{\text{F}\text{E}}\:\times\:\:{\upalpha\:},$$

where DE is the energy requirement of the fish (MJ d^− 1^) and FE is the digestible energy content in the feeds (ranging from 19.6 to 21.6 MJ kg^− 1^ d^− 1^ according to the feed used). DE was calculated according to Bureau and Hua [52] as:

4 $$\:\text{D}\text{E}=\text{R}\text{E}+\text{H}\text{e}\text{E}+\text{H}\text{i}\text{E}+\text{U}\text{E}+\text{Z}\text{E},$$

where RE is the energy retention (kJ d^− 1^), calculated as the difference in gross energy (GE) content of the carcass (kJ fish^− 1^) at initial and final body weights following Bureau et al. [53],, with GE at weight W (g) computed as:

5 $$\:\text{G}\text{E}=0.0039\:\:{\text{W}}^{2}+5.5812\:\:\text{W}.$$

The basal energy metabolism (HeE, kJ d^− 1^) was predicted as a function of water temperature (T, °C) and weight (W), using the following equation for salmonids [54, 55]:

6 $$\:\text{H}\text{e}\text{E}=(-0.01+3.26\:\:\text{T}-\:0.05\:\:{\text{T}}^{2})\:\times\:\:{\text{W}}^{0.824}.$$

Estimation of heat increment of feeding (HiE, kJ d^− 1^) was approximated as 0.60RE for rainbow trout [53].

Energy loss through urine and ammonia (non-faecal energy losses, UE + ZE, kJ d^− 1^) was estimated at 0.09(HeE + RE + HiE) for rainbow trout [53].

Assuming a constant harvest weight of 3000 g, the average DFI was set equal to 5.05 g d^− 1^ using a scaling factor α = 0.997 to achieve an FCR of 1.3 kg kg^− 1^ over the growing period in the reference scenario [36].

The third and last trait included in H was fish survival (SR). We applied a 15% mortality rate over the production cycle, from 10 g to 3000 g, assuming that the daily mortality probability varies across the rearing period and was higher in younger individuals [56]. According to Pouil et al. [36], the survival rate was modelled using a Weibull function, with the hazard function h, representing the death rate on a given day n, conditional on survival up to that point, calculated as:

7 $$\:\text{h}\left(\text{n}\right)=\:\frac{\text{f}\left(\text{n}\right)}{1-\text{F}\left(\text{n}\right)},$$

where f(n) is the Weibull density function:

8 $$f(n)=\frac{s}{\lambda}\left(\frac{n}{\lambda}\right)^{s-1}\exp(-\left(\frac{n}{\lambda}\right)^s),$$

and F(n) is the Weibull distribution function:

9 $$F(n)=1-\exp(-\left(\frac{n}{\lambda}\right)^s)$$

In our model, we kept the parameter s fixed at 0.5, while the scale parameter λ was optimized for each fish batch, to ensure a final mortality rate of 15% across the entire rearing duration [36].

To provide a biologically interpretable estimate of selection outcomes, we modelled the farm-level feed conversion ratio (FCR) after one generation of selection. For each breeding goal (H) and each scenario, we used the predicted phenotypic responses in TGC, DFI, and SR obtained from the optimal selection index calculations (see section on Environmental merit indices and expected responses to selection). These values were then used as input parameters in the farm model, which integrates growth trajectories, mortality rates, and feed requirements under standardized production conditions. The model outputs a recalculated FCR, expressed as the ratio of total feed intake to harvested biomass at the farm level, while maintaining constant total biomass production.

## Breeding goals

The breeding goal (H_X_) for each of the nine impact categories can be defined as:

10 $$\:{\text{H}}_{\text{X}}={\text{E}\text{N}\text{V}}_{\text{T}\text{G}\text{C}}^{\text{X}}\:{\text{A}}_{\text{T}\text{G}\text{C}}+\:{\text{E}\text{N}\text{V}}_{\text{D}\text{F}\text{I}}^{\text{X}}\:{\text{A}}_{\text{D}\text{F}\text{I}}+\:{\text{E}\text{N}\text{V}}_{\text{S}\text{R}}^{\text{X}}\:{\text{A}}_{\text{S}\text{R}},$$

where A_i_ is the true breeding value (or additive genetic value) for trait i; ENV_i_^X^ for trait i is calculated as the marginal impact on H_X_ (impact variation per physical unit).

This marginal impact is derived as the difference in environmental impact values before and after changing the average performance of the trait i by 0.1 physical unit, while maintaining the means of two other traits constant. We calculated the ENV in the same way from LCA for each of the nine selected impact categories X (i.e. climate change, terrestrial acidification potential, freshwater eutrophication, marine eutrophication, terrestrial ecotoxicology, freshwater ecotoxicology, land use, water dependence and CED). We thus obtained nine different breeding goals, i.e. H_CLIMATE_, H_ACID_, H_F−EUTRO_, H_M−EUTRO_, H_T−TOX_, H_F−TOX,_ H_LAND_, H_WATER,_ and H_CED_, whose responses we sought to maximize by selecting on the corresponding optimal selection indices.

### Environmental merit indices and expected responses to selection

The response to selection under each scenario was assessed by deriving an optimal selection index (I) and its accuracy. The same traits were considered in the breeding goal (H_X_) and in the selection index (I_X_) and can described as:

11 $$\:{\text{I}}_{\text{X}}=\:{\text{b}}_{\text{T}\text{G}\text{C}}^{\text{X}}\:{\text{Y}}_{\text{T}\text{G}\text{C}}+{\text{b}}_{\text{D}\text{F}\text{I}}^{\text{X}}{\text{Y}}_{\text{D}\text{F}\text{I}}+\:{\text{b}}_{\text{S}\text{R}}^{\text{X}}\:{\bar{\text{Y}}}_{\text{S}\text{R}}.$$

Where Y_TGC_ and Y_DFI_ indicate phenotypes directly measured on selection candidates for TGC and DFI, while SR is measured on half-sibs of the candidates through a collateral test. We assumed that all selection candidates have the same number of half-sibs tested in a disease challenge (hs = 23), so the phenotypic value for SR of a candidate is the average performance measured on collaterals, i.e.:

12 $$\:{\bar{\text{Y}}}_{\text{S}\text{R}}=\frac{1}{\text{h}\text{s}}\sum\:_{\text{i}=1}^{\text{h}\text{s}}{\text{S}\text{R}}_{\text{i}}.$$

The vector of coefficients (**b**_**X**_), representing the index weights for each trait of the optimal selection index [1], is given by:

13 $$\:{\mathbf{b}}_{\mathbf{X}}={\mathbf{P}}^{-1}\mathbf{G}\:{\mathbf{E}\mathbf{N}\mathbf{V}}^{\mathbf{X}},$$

where $\:{\mathbf{E}\mathbf{N}\mathbf{V}}^{\mathbf{X}}$ is the vector containing the ENV of the three traits included in H_X_; **P** is the matrix denoting the variances and covariances among the predictors Y in I, and **G** is the matrix denoting the variances and covariances between the predictors Y in I and the true additive genetic values A in H_X_. Derivation of **P** and **G** is provided in the Additional file [See Additional file 1, Text S1].

We calculated the variance of the selection index I_X_ as:

14 $$\:{{\upsigma\:}}_{\text{I}\text{x}}^{2}=\:{{\mathbf{b}}_{\mathbf{X}}}^{\mathbf{{\prime\:}}}\mathbf{P}{\mathbf{b}}_{\mathbf{X}},$$

and the genetic variance of the breeding goal (H_X_) as:

15 $$\:{{\upsigma\:}}_{\text{H}\text{x}}^{2}=\:{{\mathbf{E}\mathbf{N}\mathbf{V}}^{\mathbf{X}}}^{\mathbf{{\prime\:}}}{\mathbf{V}}_{\mathbf{H}}{\mathbf{E}\mathbf{N}\mathbf{V}}^{\mathbf{X}},$$

where **V**_**H**_ is the genetic variance-covariance matrix of traits (1,2,3) in H, i.e.:

16 $$\:{\mathbf{V}}_{\mathbf{H}}=\:\left(\begin{array}{ccc}{{\upsigma\:}}_{\text{g}1}^{2}&\:{\text{r}}_{\text{g}12}\:\sqrt{{{\upsigma\:}}_{\text{g}1}^{2}\:{{\upsigma\:}}_{\text{g}2}^{2}}&\:{\text{r}}_{\text{g}13}\:\sqrt{{{\upsigma\:}}_{\text{g}1}^{2}\:{{\upsigma\:}}_{\text{g}3}^{2}}\\\:{\text{r}}_{\text{g}12}\:\sqrt{{{\upsigma\:}}_{\text{g}1}^{2}\:{{\upsigma\:}}_{\text{g}2}^{2}}&\:{{\upsigma\:}}_{\text{g}2}^{2}&\:{\text{r}}_{\text{g}23}\:\sqrt{{{\upsigma\:}}_{\text{g}2}^{2}\:{{\upsigma\:}}_{\text{g}3}^{2}}\\\:{\text{r}}_{\text{g}13}\:\sqrt{{{\upsigma\:}}_{\text{g}1}^{2}\:{{\upsigma\:}}_{\text{g}3}^{2}}&\:{\text{r}}_{\text{g}23}\:\sqrt{{{\upsigma\:}}_{\text{g}2}^{2}\:{{\upsigma\:}}_{\text{g}3}^{2}}&\:{{\upsigma\:}}_{\text{g}3}^{2}\end{array}\right),$$

with $\:{\sigma\:}_{gj}^{2}$ the additive genetic variance of trait k and r_gjk_ the genetic correlation between traits j and k.

The accuracy of the optimal selection index (ρ_x_) was calculated as:

17 $$\:{{\uprho\:}}_{\text{x}}=\:\frac{{{\upsigma\:}}_{\text{I}\text{x}}}{{{\upsigma\:}}_{\text{H}\text{x}}}.$$

As males and females are selected with the same selection intensity and accuracy, the annual genetic change (AGC_Hx_) can be calculated as:

18 $$\:{\text{A}\text{G}\text{C}}_{\text{H}\text{x}}=\frac{{\text{i}\:{\uprho\:}}_{\text{x}}\:{{\upsigma\:}}_{\text{H}\text{x}}}{\text{L}},$$

where i is the selection intensity derived for a selection rate equal to 10% for both male and female candidates; and L is the corresponding generation interval, which was equal to 24 months.

Annual genetic change for each trait was calculated as:

19 $$\:\text{A}{\text{G}\text{C}}_{\text{X},\text{t}\text{r}\text{a}\text{i}\text{t}}=\:\frac{\text{i}}{{{\upsigma\:}}_{\text{I}\text{x}}}\:\mathbf{G}^{\mathbf{\prime\:}}{\mathbf{b}}_{\mathbf{X}}.$$

We estimated the selection accuracy (ρ_x_) and annual genetic changes (AGC_Hx_) for the breeding goals corresponding to the different impact categories. To assess the consequences of selection to maximize response on a given breeding goal H_x_ on responses for the other impact categories (Y ≠ X), we calculated the annual indirect selection responses (R_Y_) on H_Y_ when the optimal selection is I_X_ as:

20 $$\:{\text{R}}_{\text{Y}\ne\:\text{X}}=\sum\:{\text{E}\text{N}\text{V}}_{\text{t}\text{r}\text{a}\text{i}\text{t}}^{\text{Y}}\:{\text{A}\text{G}\text{C}}_{\text{X},\:\text{t}\text{r}\text{a}\text{i}\text{t}}.$$

### Genetic parameters

The genetic parameters for the three traits are in Tables 1 and 2. They were derived from the literature on rainbow trout. Reported heritability (h²) values ranged from 0.12 to 0.32 for TGC across different ages [57–59], from 0.03 to 0.19 for DFI at various ages and under different diets [60–62], and from 0.08 to 0.17 for SR at different ages [58, 63, 64].

**Table 1:** Phenotypic means, coefficients of phenotypic variation (CV_P_), and heritability (h^2^) for thermal growth coefficient (TGC), daily feed intake (DFI), and survival rate (SR)

Trait	Mean	CV_P_ (%)		h^2^	References
		Low*	High*		
TGC (g^1/3^ °C^−1^ d^−1^)	1.80	10	25	0.22	[58–60]
DFI (g d^−1^)	5.05	20	50	0.10	[61–63]
SR (%)	85	42**	42**	0.10	[59, 64, 65]

*Given the variations observed for TGC and DFI in the literature according to the age and/or diet of the fish [53], we considered two scenarios for CV_P_ of these two traits

**Estimated from the phenotypic variance: Vp = µ_P_ × (1 − µ_P_)

**Table 2:** Scenarios A and B of genetic and phenotypic correlations between thermal growth coefficient (TGC), daily feed intake (DFI), and survival rate (SR)

Traits	Genetic correlation		Phenotypic correlation	
	A	B	A	B
TGC-DFI	0.90	0.90	0.60	0.60
TGC-SR	0	0.60*	0	0
DFI-SR	0	0.54*	0	0

*We considered two scenarios considering (scenario B) or not (scenario A) genetic correlations SR and the other traits as suggested by Sae-Lim et al. [58] showing genetic correlation between SR and TGC in pan-size trout and assuming that r_g(DFI,SR)_ can be estimated as r_g(TGC,SR)_ × r_g(TGC,DFI)_

Genetic standard deviations (σ_g_) were calculated as:

21 $$\:{{\upsigma\:}}_{\text{g}}=\:\sqrt{{\text{h}}^{2}\:{{\upsigma\:}}_{\text{p}}^{2}},$$

where σ_p_^2^ is the phenotypic variance of the trait.

Given the variations observed for TGC and DFI in the literature according to the age and/or diet of the fish [60], σ_p_^2^ was calculated using two sets of coefficients of variation CV_p_ (%): 10 and 20 for TGC and DFI, respectively (scenario “Low”), or 20 and 50 for TGC and DFI, respectively (scenario “High”), and based on the phenotypic mean of the traits of 1.8 g^1/3^ d^− 1^ °C^− 1^ and 5.05 g d^− 1^ for TGC and DFI, respectively, Table 1). For SR, σ_p_^2^ = p × (1 − p) was estimated using mortality of 15% in the hypothetical farm.

Based on published estimates, the genetic correlation between TGC and DFI was assumed to be 0.9, while the phenotypic correlation was set at 0.6 [62]. Due to discrepancies in the literature, we considered two scenarios for genetic correlations between SR and other traits. Sae-Lim et al. [50] reported a positive genetic correlation (rg) between SR and TGC in pan-size trout, but this correlation has not been consistently observed. Therefore, in Scenario A, rg(TGC-SR) was assumed to be zero, while rg(TGC-SR) was positive in the Scenario B. For the latter, we estimated rg(DFI-SR) as the product of rg(TGC-SR) and rg(TGC-DFI). Phenotypic correlations between SR and the other traits were consistently assumed to be zero (Table 2).

## Results

### Environmental impacts and contribution analysis

**Fig. 2:**
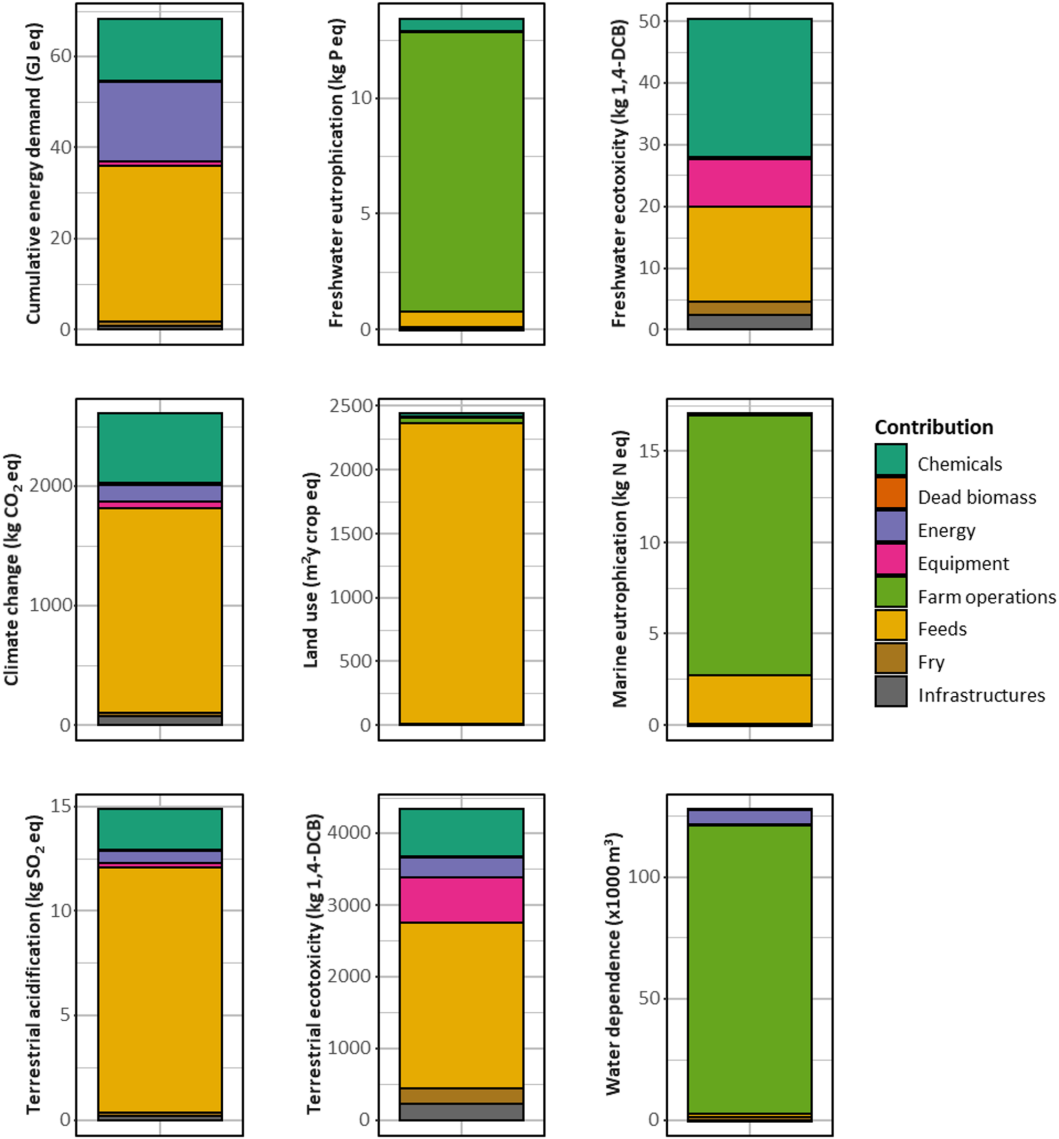
Environmental impacts of rainbow trout production at the level of the hypothetical farm. The assessment of the impacts was carried out using ReCiPe 2016 Midpoint (H) version 1.07 considering nine impact categories

Figure 2 presents the level of environmental impacts of conventional production of rainbow trout and the contribution of system components to the impacts. Feed (i.e., feed production, milling, and transport) was among the main contributors to the environmental impacts related to land use (97% of 2443 m^2^y crop eq), terrestrial acidification (79% of 14.9 kg SO_2_ eq), climate change (66% of 2602 kg CO_2_ eq), terrestrial ecotoxicity (53% of 4334 kg 1,4-DCB eq), and CED (50% of 68 GJ). Farm operations (i.e., farm operations and on-farm emissions) was the main contributor to water dependence (93% of 127 10^3^ m^3^), terrestrial (90% of 13.4 kg P eq), and marine eutrophication (84% of 17.1 kg N eq). Interestingly, the chemicals used were primarily liquid oxygen, while antibiotics, other veterinary products, and disinfectants were very low contributors to the impacts. Thus, liquid oxygen represented between 13 and 44% of the impacts for CED, freshwater and terrestrial ecotoxicity, climate change, and terrestrial acidification potential (Fig. 2). Electricity consumption was the main contributor to the CED (26% of 68 GJ eq), while equipment accounted for 0 to 15% of the environmental impacts at the farm level depending on the impact category. Dead biomass, fry, and infrastructures had a negligible contribution (< 5%) on each impact category (Fig. 2).

### ENV derivation

LCA was used to estimate ENV, which were then applied as weights in the three-trait breeding goals. The ENV estimates from various impact categories are shown in Table 3. For SR, the relative weight (RW, %) of the ENV was low for most breeding goals (H_X_) for all coefficient of variation (CV_p_) scenarios, generally staying below 2%. Relative weights for TGC and DFI, denoted as RW_TGC_ and RW_DFI_, respectively, remained fairly consistent for most H_X_ scenarios. The highest RWs were consistently found for DFI, except for H_WATER_, ranging from 84.2% for H_T−TOX_ in the “Low” scenario to 86.3% for H_CED_ in the “High” scenario. Meanwhile, RW_TGC_ ranged from 13.6% for H_CED_ in the “Low” scenario to 15.5% for H_T−TOX_ in the “High” scenario. H_WATER_ stood out from the other breeding goals with distinct RW patterns. For all H_X_, RWs followed decreased in the order: RW_SR_ < < RW_TGC_ < < RW_DFI_. Due to similar weighting patterns across H_X_, we focused on three breeding goals for further analysis: H_F−EUTRO_, H_T−TOX_, and H_WATER_ (Table 3).

**Table 3:** Environmental values (ENV) for the thermal growth coefficient (TGC), daily feed intake (DFI), and survival rate (SR) expressed in physical units and relative weights of the ENV (RW, %) in the breeding goal (H), with traits standardized by their genetic standard deviation considering the two scenarios for coefficients of variation (CV_P_), as specified in Table 1

Impact category	Breeding goal name	ENV (physical units)			RW (%) – Low CV_P_			RW (%) – High CV_P_		
		ENV_TGC_	ENV_DFI_	ENV_SR_	RW_TGC_	RW_DFI_	RW_SR_	RW_TGC_	RW_DFI_	RW_SR_
Climate change (kg CO_2_ eq)	H_CLIMATE_	− 350.1	567.0	− 5.318	13.99	85.72	0.29	14.02	85.87	0.11
Terrestrial acidification potential (kg SO_2_ eq)	H_ACID_	− 1.877	3.082	− 0.02386	13.84	85.92	0.24	13.85	86.05	0.10
Freshwater eutrophication (kg P eq)*	**H**_**F**_ − _**EUTRO**_	− **2.113**	**3.502**	− **0.02104**	**13.73**	**86.04**	**0.23**	**13.75**	**86.18**	**0.07**
Marine eutrophication (kg N eq)	H_M_ − _EUTRO_	− 3.160	5.178	− 0.02561	13.87	85.98	0.15	13.88	86.06	0.06
Terrestrial ecotoxicology (kg 1,4-DCB)*	**H**_**T-TOX**_	− **493.3**	**710.9**	− **7.381**	**15.45**	**84.24**	**0.31**	**15.48**	**84.40**	**0.12**
Freshwater ecotoxicology (kg 1,4-DCB)	H_F-TOX_	− 7.181	11.89	− 0.08567	13.74	86.04	0.22	13.76	86.16	0.08
Land use (m^2^ yr^−1^ crop eq)	H_LAND_	− 289.2	473.0	− 4.032	13.88	85.86	0.26	13.90	86.00	0.10
Water dependence (m^3^)*	**H**_**WATER**_	− **470.9**	**245.3**	− **18.28**	**33.08**	**65.20**	**1.72**	**33.43**	**65.88**	**0.69**
Cumulative energy demand (GJ)	H_CED_	− 7.207	12.12	− 0.08359	13.56	86.23	0.21	13.58	86.34	0.08

*In bold are indicated the breeding goals selected for the next analysis steps: H_F-EUTRO_, H_T-TOX_ and H_WATER_

### Annual genetic changes

Overall, selection accuracy ranged from 0.34 to 0.43. The lowest accuracy was observed for the breeding goal H_WATER_, ranging from 0.34 to 0.35, whereas accuracy remained consistently high for H_F−EUTRO_ and H_T−TOX_, ranging from 0.41 to 0.43 (Table 4). Annual genetic changes expected for the breeding goals (AGC_H_), expressed per ton of trout produced at the farm level, ranged from -0.35 to -0.91 kg P eq for H_F−EUTRO_, from -68.7 to -179.5 kg 1,4-DCB for H_T−TOX_ for terrestrial ecotoxicity, and from -11.9 to -56.1 m^3^ for H_WATER_.

**Table 4:** Predictions of index accuracy and annual genetic changes (AGC) in the breeding goal and traits included

Prediction	Units	Scenarios			
		Low CV_P_		High CV_P_	
		A	B	A	B
H_F-EUTRO_					
Accuracy		0.41	0.43	0.41	0.43
AGC_H_	kg P eq	− 0.35 (− 2.61%)	− 0.36 (− 2.72%)	− 0.87 (− 6.52%)	− 0.91 (− 6.80%)
AGC_TGC_	σ_g_	− 0.40	− 0.42	− 0.40	− 0.42
AGC_DFI_	σ_g_	− 0.38	− 0.39	− 0.38	− 0.39
AGC_SR_	σ_g_	+ 0.01	− 0.13	+ 0.01	− 0.13
H_T-TOX_					
ρ_H_		0.41	0.43	0.41	0.43
AGC_H_	kg 1,4-DCB	− 68.7 (− 1.59%)	− 71.9 (− 1.66%)	− 171.9 (− 4.00%)	− 179.5 (− 4.14%)
AGC_TGC_	σ_g_	− 0.40	− 0.42	− 0.40	− 0.42
AGC_DFI_	σ_g_	− 0.38	− 0.39	− 0.38	− 0.39
AGC_SR_	σ_g_	+ 0.01	− 0.13	+ 0.01	− 0.13
H_WATER_					
ρ_H_		0.34	0.35	0.34	0.35
AGC_H_	m^3^	− 11.9 (− 0.01%)	− 33.8 (− 0.03%)	− 33.6 (− 0.03%)	− 56.1 (− 0.04%)
AGC_TGC_	σ_g_	− 0.38	− 0.40	− 0.38	− 0.40
AGC_DFI_	σ_g_	− 0.37	− 0.38	− 0.37	− 0.39
AGC_SR_	σ_g_	+ 0.01	− 0.12	+ 0.01	− 0.12

(1) expressed in physical units for the three breeding goals ( H_F-EUTRO_, H_T-TOX_ and H_WATER_) (in brackets expressed as % of the mean farm environmental impact)and (2) expressed in genetic standard deviation (σ_g_) for the three traits (TGC, DFI and SR) according to two scenarios for CV_P_ (i.e. “Low” and “High”; see Table 2) and two scenarios for genetic correlations (r_g_) between traits (i.e. A and B; see Table 3)

To assess the significance of such gains in relation to the environmental impacts for the hypothetical farm before selection, we calculated the annual relative changes in impacts based on the optimal selection indices (Table 4). These relative changes, expressed as percentage reductions, ranged from -0.01 to -6.80% for the different breeding goals. The largest reductions were observed for H_F−EUTRO_ (i.e., -2.61 to -6.80%), whereas the breeding goal targeting water dependence reduction (H_WATER_) resulted in minimal reductions, from -0.01 to 0.04%. The magnitude of these changes depended on the CV for TGC and DFI, with the smallest reductions observed in the “Low” scenario and the largest reductions in the “High” scenario.

When examining genetic changes for each trait, similar changes were achieved for TGC and DFI for the three H. Given the high genetic correlations between these two traits, improvement in DFI by 0.37 to 0.39 genetic standard deviations (σ_g_) was expected to be accompanied by the observed decrease in TGC, ranging from -0.38 to -0.42 σ_g_ for these two breeding goals. Notably, these genetic changes effectively translate into reductions in FCR ranging from 1.7 to 4.8% (Table 5). Regardless of the breeding goal considered, AGC_SR_ were mostly negative or close to zero, with responses ranging from -0.13 to +0.01 σ_g_. The most unfavourable response to selection was obtained for the strongest genetic correlations evaluatred (scenario B) (Table 4).

**Table 5:** Predictions of indirect annual genetic gains in feed conversion ratio (FCR) expressed in physical unit (kg feeds kg biomass^−1^) for the three breeding goals (H_F-EUTRO_, H_T-TOX_ and H_WATER_)

Breeding goal	FCR unit	Scenarios			
		Low CV_P_		High CV_P_	
		A	B	A	B
H_F-EUTRO_	kg feeds kg biomass^−1^	− 0.025 (1.9%)	− 0.023 (1.7%)	− 0.062 (4.8%)	− 0.061 (4.8%)
H_T-TOX_	kg feeds kg biomass^−1^	− 0.025 (1.9%)	− 0.023 (1.7%)	− 0.062 (4.8%)	− 0.061 (4.7%)
H_WATER_	kg feeds kg biomass^−1^	− 0.025 (1.9%)	− 0.023 (1.8%)	− 0.061 (4.7%)	− 0.061 (4.7%)

In brackets, the gains expressed as % of the reference FCR of the hypothetical farm (i.e. 1.293 kg kg^−1^). We considered the two scenarios for CV_P_ ( “Low” and “High”; see Table 2) and two scenarios for genetic correlations (r_g_) between traits (A and B; see Table 3)

**Fig. 3:**
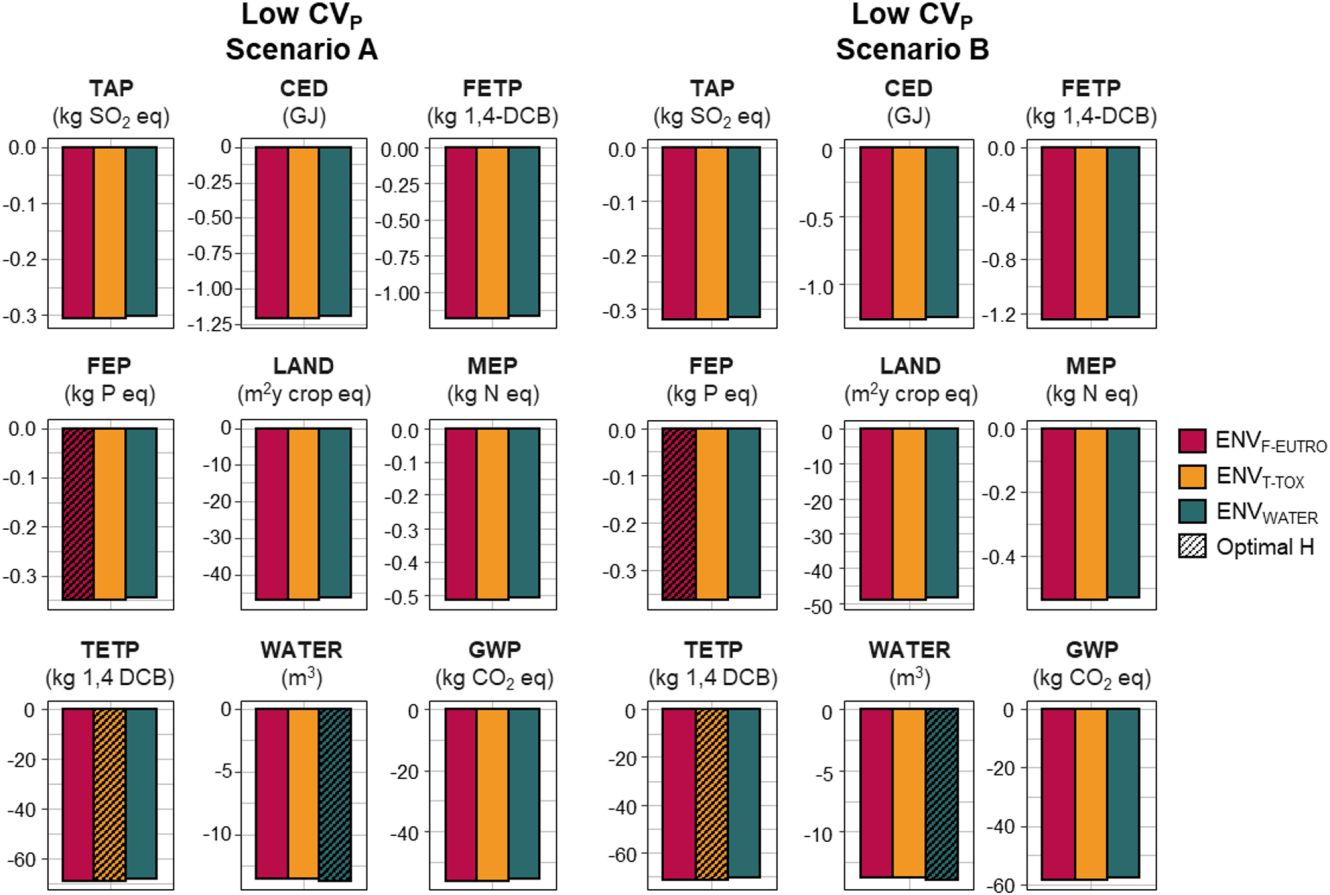
Annual gains (in physical units) for the different impact categories. TAP: terrestrial acidification, CED: Cumulative energy demand, FETP: freshwater ecotoxicity, FEP: freshwater eutrophication, LAND: land use, MEP: water dependence, TETP: terrestrial ecotoxicity, WATER: water dependence and GWP: climate change according to the “Low” CV_p_ scenario; see Table 2 and two scenarios of genetic correlations (rg) between traits (i.e. A and B; see Table 2). Responses on H_X_ for the optimal selection index I_X_ for each impact category are black dashed.

Figure 3 illustrates the genetic gains across all nine impact categories, depending on which category was prioritized as the breeding goal (i.e., the impact used to define the optimal selection index). We focused on comparing the two scenarios of genetic correlations, assuming low CV_p_ for TGC and DFI as the most realistic scenario. Overall, selecting for the any of the breeding goals led to similar reductions in environmental impacts, with relatively small differences arising from setting genetic correlations between SR and other traits to zero or not. Notably, selection based on these breeding goals effectively reduced environmental impacts across all categories considered in this study.

As expected, the genetic change for a given environmental impact was reduced when selection was based on an index optimized for a different impact category. However, this reduction remained modest - less than 2% compared to the optimal index - regardless of the genetic correlation scenario considered (Scenario A or B).

## Discussion

### Integrating environmental objectives into breeding goals

To our knowledge, this study is the first to predict expected selection responses in a breeding program designed to minimize the environmental impacts of rainbow trout farming. The study focused on the production of large rainbow trout (3 kg at harvest), modelled through a hypothetical farm based on surveys conducted among French fish farmers [36]. Several breeding goals were examined, each including three key traits of interest, weighted by their respective ENV. These ENV quantify the marginal changes in environmental impacts resulting from improvements in trait performance. They were derived from nine LCA impact categories previously identified as the primary environmental concerns in rainbow trout production systems [30, 32–37]. A cradle-to-farm-gate LCA was adopted to derive the ENV of the traits of interest. This approach considers not only the environmental impacts occurring at the farm level but also those associated with the production of farm inputs. While the fish farming sector is not directly accountable for mitigating the environmental impacts of industrial processes involved in producing farm inputs, including these off-farm impacts in the analysis is crucial, as ignoring these emissions could lead to strategies that appear to reduce environmental impacts at the farm level but inadvertently increase impacts during the production of farm inputs [23]. Moreover, by considering environmental impacts alongside cost, fish farmers can make more informed choices when selecting inputs.

The three primary traits analysed in this study, growth rate measured as TGC, DFI, and SR, were identified as priorities by farmers through surveys. These traits have also been emphasized in previous research on breeding preferences among rainbow trout farmers. For example, Sae-Lim et al. [6] ranked SR, FCR, and TGC as the most important traits across 53 rainbow trout farmers surveyed across continents, production systems (RAS or flow-through), and market products (fry, pan-sized, or large fish), using an analytic hierarchy process combined with weighted goal programming to aggregate the farmers’ preferences to consensus preference values. The relative contribution of these three traits to the breeding goals varied depending on the targeted environmental impact category. Notably, DFI had a substantial influence on all breeding goals, particularly for reducing eutrophication and land competition. In contrast, when selection focused on reducing water dependence, the relative ENV of DFI (ENV_DFI_) was slightly lower, shifting to a higher contribution of TGC (ENV_TGC_) in this breeding goal. Across the breeding goals considered, the estimated ENV of SR (ENV_SR_) was consistently low.

### Relative trait contributions across environmental impact categories

To understand the relative ENV, it is essential to analyse the consequences of an improvement in the performance of the three traits, while maintaining constant annual production at the farm scale. For SR, an improvement primarily results in a reduction in the number of eggs required at the start of the production cycle, thereby slightly lowering environmental impacts at this stage. A similar logic applies to DFI, where a lower in DFI leads to a reduction in feed inputs, while the environmental impacts of other production factors remain constant. As reviewed by Bohnes et al. [45], feed represents the major contributor to environmental impacts of aquaculture systems. Logically, ENV_DFI_ was higher compared to the two other traits in all breeding goals. These findings are consistent with previous studies on other fish species such as African catfish (*Clarias gariepinus*) [26] and European seabass [25, 65], which highlighted the central role of feed efficiency in reducing acidification, climate change, CED, and eutrophication. We found that the RW of ENV_DFI_ was the highest for H_F−EUTRO_ and H_CED_, partially aligning with the findings of Besson et al. [26]. Their study demonstrated that among the four evaluated impact categories, the highest ENV (expressed as a percentage per tonne of fish) for FCR in African catfish reared in RAS were associated with eutrophication but they found lower ENV for CED than we did.

In contrast, the effects of improving TGC on the farm’s overall environmental impacts are more complex to understand. An improvement in growth rate, under the constraint of constant production, leads to a reduction in rearing times, which subsequently affects the use of rearing infrastructure and equipment. The extent of these changes, however, depends on environmental factors such as temperature and water availability. For instance, faster early growth rates influence the utilization of water recirculation systems and oxygen supply equipment but this is seasonally dependent: during low-water periods, the effects are more pronounced, whereas in winter, when water is abundant and temperatures are low, the need for water recirculation and oxygen supply is the lowest. Overall, it is not surprising that the relative weight of ENV_TGC_ is always intermediate between those of DFI and SR. However, it doubles when selection focuses on reducing water dependence (H_WATER_). In African catfish reared in RAS, Besson et al. [26] found that the highest ENV_TGC_ were for acidification, followed by CED and climate change, while ENV_TGC_ was much lower for eutrophication. In our study, the RW of ENV_TGC_ in the breeding goals was approximately the same for acidification, CED, and climate change. The impact of changes in TGC at the level of the farm is influenced by the production system (open flow-through or RAS) and the associated limiting factors. In their study, Besson et al. [26] considered two types of limiting factors: maximum stocking density and maximum dissolved NH_3_-N concentration. When dissolved NH_3_-N was the limiting factor, improvements in TGC did not enhance production or production efficiency. However, when stocking density was the limiting factor, improving TGC increased production, thereby diluting fixed environmental impacts over a larger number of fish and the environmental impacts per tonne of fish produced decreased. These differences in system constraints may explain the differences in the magnitude of the ENV_TGC_ between our study and that of Besson et al. [26].

The low ENV estimated for survival (SR) in our study contrasts with previous findings, such as those reported by Johansen et al. [66] in salmon farming, where mortality had a notable impact on carbon emissions due to wasted feed. This discrepancy likely stems from key differences in modelling assumptions. In our approach, the system was constrained to a constant harvest biomass, meaning that improvement in survival primarily reduced the number of juveniles required, but had only a limited effect on overall feed intake and infrastructure use. As a result, the environmental benefit of improving SR was lower than in systems where mortality directly increases total input use. This highlights the importance of production system assumptions when interpreting ENV and suggests that SR could have a higher environmental relevance in more input-sensitive contexts.

### Genetic responses and trade-offs between traits

Given the similarities observed in the ENV, we decided to focus on breeding goals aimed at reducing environmental impacts across three key impact categories among the nine initially assessed: H_F−EUTRO_, H_T−TOX,_ and H_WATER_. Our findings show that selection strategies aimed at reducing environmental impacts can be effective, although their effectiveness varied depending on the targeted impact category. In particular, for H_WATER_, we found that annual genetic changes were remarkably low, with reductions in water dependence remaining below 0.1% per year. This outcome demonstrates that such a selection strategy is ineffective for targeting this impact category, regardless of the level of CV_p_ or the intensity of genetic correlations between the traits of interest. Selecting for reduced freshwater eutrophication is expected to lower this impact by 2.6–6.8% per year, depending on the scenarios considered, while terrestrial ecotoxicity can be reduced by 1.6 to 4.1%. Such results are even better than the 0.9 to 2.7% annual reduction in eutrophication reported by Besson et al. [25] for European seabass when considering a breeding goal combining TGC and FCR weighted by ENV, despite the fact that we used a lower heritability and genetic variances for both TGC and DFI. The magnitude of this difference can reasonably be explained by the differences in weights between the traits used in the breeding goals. Selection based on any of the three breeding goals enabled an annual improvement in DFI (i.e. a reduction in feed intake) ranging from -0.40 to -0.37 σ_g_. However, due to the strong negative genetic correlation of DFI with TGC, growth rate was adversely affected. The consequence of lowering TGC was an increase in production cycle time. In our model, we estimated that, for the “Low” CV scenario, the reduction in TGC could increase time to reach 3 kg by up to 6% per year. In a commercial setting, such a delay may be economically unfavourable. Nonetheless, it is noteworthy that FCR decreased annually by 1.7 to 1.9 under the same CV scenario. This improvement could lead to a reduction of up to 40% after 10 generations of selection (20 years), representing an improvement twice as large as that reported by Vandeputte et al. [67], who calculated realized genetic gains in growth, survival, FCR, and quality traits in multi-trait selection. This difference can be attributed primarily to the focus of their selection program on fillet production (improving growth, carcass yield, and fillet fat), with the improvement in FCR being an indirect outcome of selection.

Responses to selection were conditioned both by the magnitude of the phenotypic variability for the traits considered in the selection indexes and the genetic and phenotypic correlations between these traits. Given the different trait parameter estimates reported in the literature depending on age and/or diet of the fish [60], we assumed two scenarios of CV_p_ for TGC and DFI. Logically, the scenario with the greatest phenotypic variability resulted in the highest AGC. However, the CV_p_ we relied on - sourced from the literature - were not adjusted for all systematic environmental effects, unlike the corrected phenotypes typically used in breeding programs [68]. Therefore, it is reasonable to assume that the scenario with the lowest CV_p_ for these two traits is the most realistic representation.

In rainbow trout, and fish species more broadly, significant uncertainty remains regarding the magnitude of genetic correlations between some traits, despite an increasing number of studies on the topic. Most research to date has focused primarily on growth and yield-related traits (e.g [69, 70]). Faced with some discrepancies in previous studies and the lack of data, we explored scenarios with or without genetic correlations between SR and the other traits set to zero. Overall, our results demonstrate that selection responses were generally not strongly influenced by the strength of genetic correlations between traits. The main exception was the response in SR, which was negative when it had a positive genetic correlation with TGC and DFI. The genetic correlation of approximately 0.60 between SR and TGC estimated by Sae-Lim et al. [58] was observed only within a size range of trout between 27 and 376 g over a rearing period of about eight months. In contrast, in our study, 10-g juvenile trout reached a harvest weight of 3000 g after approximately 24 months. Vehviläinen et al. [71] demonstrated that the nature and strength of genetic correlations between SR and weights of rainbow trout depend on the developmental stage and rearing system, with estimates ranging from -0.14 to +0.40. These results suggest that a zero genetic correlation is likely when rearing trout from 10 g to 3000 g, the harvest size typically targeted in the current French production [72]. Nevertheless, even in the absence of genetic correlations with the other traits, SR was not substantially improved for any of the nine breeding goals, with AGC_SR_ limited to 0.01 σ_g_. These findings highlight the limitations of an optimal index that combines these traits to reduce environmental impacts and raise concerns about its relevance on economic and social perspectives. We also acknowledge that breeders and farmers may value genetic gains differently from the perspective of consensus preferences.

### Implications for sustainable breeding strategies

By considering a scenario deemed to have the most realistic assumptions for CV_p_ and genetic correlations between traits, we estimated AGC across the nine environmental impact categories relative to each of the selected breeding goals. This approach enabled us to assess the indirect effects of selection strategies on other environmental impact categories. As expected, AGC for a given environmental impact category diminished when the optimal selection index for that category was not applied, although these reductions greatly depended on the index used. Among the breeding goals, selection targeting reduced water dependence was slightly less effective in achieving gains across other impact categories. However, the differences remained minimal, with environmental impact reductions in non-target categories consistently within 2% of the optimal scenario. This suggests that, despite some trade-offs, using any of the environmentally oriented selection indices would still lead to substantial improvements across all environmental impacts. These findings support the robustness of environmentally weighted breeding goals and provide flexibility for breeders to prioritize impacts based on specific policy or production contexts.

In practice, sustainable aquaculture breeding programs require a unified composite breeding goal that balances multiple, often competing, objectives. While our study analyzed environmental impacts separately, a meaningful integration into classical selection index theory necessitates expressing all components using a common unit. As emphasized by Olesen et al. [15], this typically implies translating all objectives - whether economic or environmental - into a shared economic scale. In this context, ENV derived from LCA could be converted into economic terms, for example, by applying marginal abatement costs or market-based carbon pricing schemes. This allows direct comparison and aggregation with EV, and facilitates the construction of a combined index. Such a unified index could either optimize economic returns under explicit environmental constraints or, conversely, minimize environmental burdens subject to economic viability thresholds - depending on the priorities of the breeding program.

Besson et al. [25] illustrated the relevance of this approach, showing that selection for feed efficiency (FCR) simultaneously improves both economic and environmental performance, whereas selection for growth (TGC) may lead to trade-offs, particularly under resource-intensive production scenarios. Furthermore, when EV and ENV point in conflicting directions for specific traits, one practical strategy is to implement constrained optimization or independent culling levels, for instance, by enforcing a minimum threshold for such traits, while ranking candidates on a multi-trait index for other traits.

Several complementary methodologies are also available to support the weighting of heterogeneous objectives. The desired gains approach allows breeders to set predefined trade-offs between production and other traits [9, 16, 17]. Contingent valuation and choice experiments can provide monetary proxies for social preferences regarding sustainability or welfare traits [15, 18]. Finally, participatory multi-criteria approaches, such as analytic hierarchy processes, have been applied to rainbow trout to reach consensus on breeding objectives among stakeholders with diverse priorities [6]. In the latter study, they estimated desired genetic gains expressed as percentages of the mean (1.63% for SR, 1.87% for FCR, and 1.67% for TGC). Our analysis suggests that, from an environmental perspective, genetic gains in TGC should ideally be avoided, whereas farmers often assign it a high priority. This divergence illustrates the importance of being able to evaluate DFI directly, so that breeders may better reconcile environmental goals with farmer expectations and potentially reach a consensus. Future research should explore the long-term genetic trajectories and sustainability outcomes of such integrated, constraint-aware selection strategies.

## Conclusions

This study demonstrated how weighting traits by ENV in breeding goals can reduce different environmental impacts in rainbow trout farms, while maintaining genetic improvements, notably, in DFI and, as a consequence, in FCR. However, careful attention is required to avoid compromising SR. The notable variations in selection efficiency across different targeted impact categories highlight the potential value of prioritizing specific environmental impacts in selection strategies based on ENV. Our approach opens new possibilities for developing selection indices in fish breeding programs that incorporate additional trait weighting strategies, reflecting societal priorities such as sustainability, ethics, and animal welfare.

## Supplementary Information

Below is the link to the electronic supplementary material.

Additional file 1. Derivation of P and G matrices.

## Data Availability

The datasets and R code generated during the current study are not publicly available while the authors continue to perform additional analyses and software developments. They may be available from the corresponding author upon reasonable request.
