# Supplementary material for: Deriving breeding goals and expected selection responses to reduce environmental impacts in rainbow trout farming
Source: Genet Sel Evol. 2025 Dec 13;57:71. doi: 10.1186/s12711-025-01018-5 (PMC12715902; doi:10.1186/s12711-025-01018-5)
Supplement: Supplementary file 1 — Additional file 1. Derivation of P and G matrices. [file 12711_2025_1018_MOESM1_ESM.docx]

**Additional file 1**

For the P-matrix:

Information sources: $Y_{1}$ = performances on candidates (z) for TGC

$Y_{2}$ = performances on candidates (z) for DFI

$\bar{Y}_{3}$ = mean performance from 23 half-sibs (*hs*) for SR

| $Var\left( Y_{1} \right)= \sigma_{p1}^{2}$  $Var\left( Y_{2} \right)= \sigma_{p2}^{2}$ | for the phenotypic variance of measurements of TGC and DFI on candidates |
| --- | --- |
| $Var\left( \bar{Y_{3}} \right)= \frac{\sigma_{p3}^{2}+ \frac{1}{4} \sigma_{a3}^{2} hs-1}{nhs}$ | for the phenotypic variance of a mean of SR records from n = 23 half-sibs (hs) per candidate |
| $Cov\left( Y_{1},Y_{2} \right)= r_{p12} \sqrt{\sigma_{p1}^{2} \sigma_{p2}^{2}}$ | where $r_{p12}$ is the phenotypic correlation between TGC and DFI |
| $Cov\left( Y_{1}, \bar{Y}_{3} \right)= \frac{1}{4}r_{a13} \sqrt{\sigma_{a1}^{2} \sigma_{a3}^{2}}$ | where $r_{a13}$ is the genetic correlation between TGC and SR |
| $Cov\left( Y_{2}, \bar{Y}_{3} \right)= \frac{1}{4}r_{a23} \sqrt{\sigma_{a2}^{2} \sigma_{a3}^{2}}$ | where $r_{a23}$ is the genetic correlation between DFI and SR |

$\boldsymbol{P}=\left( \begin{matrix} Var\left( Y_{1} \right) & Cov\left( Y_{1},Y_{2} \right) & Cov\left( Y_{1},\bar{Y}_{3} \right) \\ Cov\left( Y_{2},Y_{1} \right) & Var\left( Y_{2} \right) & Cov\left( Y_{2},\bar{Y}_{3} \right) \\ Cov\left( \bar{Y}_{3},Y_{1} \right) & Cov\left( \bar{Y}_{3},Y_{2} \right) & Var\left( \bar{Y_{3}} \right) \end{matrix} \right)$

For the G-matrix:

| $Cov\left( Y_{1}, A_{1} \right)= \sigma_{a1}^{2}$  $Cov\left( Y_{2}{,A}_{2} \right)= \sigma_{a2}^{2}$ | for the genetic variance of measurements of TGC and DFI on candidates |
| --- | --- |
| $Cov\left( \bar{Y_{3},}A_{3} \right)=\frac{1}{4}\sigma_{a3}^{2}$ | for the genetic variance of a mean of n records of SR from n = 23 half-sibs per candidate |
| $Cov\left( Y_{1},A_{2} \right)= r_{a12} \sqrt{\sigma_{a1}^{2} \sigma_{a2}^{2}}$ | where $r_{a12}$ is the genetic correlation between TGC and DFI |
| $Cov\left( Y_{1},A_{3} \right)= {\frac{1}{4} r}_{a13} \sqrt{\sigma_{a1}^{2} \sigma_{a3}^{2}}$ | where $r_{a13}$ is the genetic correlation between TGC and SR |
| $Cov\left( Y_{2},A_{3} \right)= \frac{1}{4} r_{a23} \sqrt{\sigma_{a2}^{2} \sigma_{a3}^{2}}$ | where $r_{a23}$ is the genetic correlation between DFI and SR |

$$\boldsymbol{G}=\left( \begin{matrix} Cov\left( Y_{1},A_{1} \right) & Cov\left( Y_{1},A_{2} \right) & Cov\left( Y_{1},A_{3} \right) \\ Cov\left( Y_{2},A_{1} \right) & Cov\left( {Y_{2},A}_{2} \right) & Cov\left( Y_{2},A_{3} \right) \\ Cov\left( Y_{3},A_{1} \right) & Cov\left( Y_{3},A_{2} \right) & Cov\left( {Y_{3}, A}_{3} \right) \end{matrix} \right)$$
